# The usefulness of bone SPECT/CT imaging with volume of interest analysis in early axial spondyloarthritis

**DOI:** 10.1186/s12891-015-0465-x

**Published:** 2015-02-04

**Authors:** Yong-il Kim, Minseok Suh, Yu Kyeong Kim, Ho-Young Lee, Kichul Shin

**Affiliations:** Department of Nuclear Medicine, Seoul National University Bundang Hospital, 82, Gumi-ro 173 Beon-gil, Bundang-gu, Seongnam-si, Gyeonggi-do 463-707 South Korea; Department of Molecular Medicine and Biopharmaceutical Sciences, WCU Graduate School of Convergence Science and Technology, Seoul National University, Seoul, South Korea; Department of Nuclear Medicine, Seoul National University Hospital Boramae Medical Center, Seoul, South Korea; Cancer Research Institute, Seoul National University, Seoul, South Korea; Department of Internal Medicine, Seoul Metropolitan Government-Seoul National University Hospital Boramae Medical Center, 425 Shin-dae-bang Dong, Dong-jak Gu, Seoul, 156-707 South Korea

**Keywords:** SPECT/CT, Bone scintigraphy, Sacroiliitis, SIS ratio, Axial spondyloarthritis

## Abstract

**Background:**

The role of conventional bone scintigraphy in diagnosing early axial spondyloarthritis (SpA) is yet controversial. Single positron emission computed tomography (SPECT) plus CT is an imaging modality that adds better anatomical information to scintigraphy of the sacroiliac (SI) joint. Our aim was to investigate the usefulness of bone SPECT/CT with volume of interest (VOI) analysis in early axial SpA patients.

**Methods:**

Twenty patients (male: female ratio = 12:8; age range = 17–65 years) presenting with inflammatory back pain meeting the Amor criteria of early axial SpA were recruited from a single center in South Korea. Bone scintigraphy was performed 180 min after intravenous injection of 1110 MBq of Tc-99 m-HDP, followed by bone SPECT/CT. The ratio between the entire SI joint and sacrum (SIS ratio) was measured by both bone SPECT/CT and bone scintigraphy. Data from 13 controls were also evaluated. Receiver operating characteristic (ROC) curve was plotted for further analysis, and the correlation between the SIS ratio and SI joint grade by plain radiography was assessed.

**Results:**

The SIS ratio of early axial SpA patients vs. control subjects was significantly increased in bone SPECT/CT (*p* < 0.001). However, no significant difference was detected in bone scintigraphy. ROC curve analysis showed a significant difference in the area under curve (AUC) of bone SPECT/CT vs. bone scintigraphy (0.862 *vs.* 0.523, respectively; *p* < 0.001). With a cut-off SIS ratio of 1.50, ROC curve analysis showed a sensitivity of 80.0% and specificity of 84.6% in bone SPECT/CT. The SIS ratio measured in SPECT/CT, but not that measured in bone scintigraphy, was significantly increased with a higher grade of SI joint changes in plain radiography (*p* = 0.014).

**Conclusion:**

Bone SPECT/CT is more useful than conventional bone scintigraphy in identifying sacroiliitis in early axial SpA patients, even with mild SI joint changes in plain radiography. By combining CT, we can accurately delineate the sacrum and SI joint uptake with our VOI method.

## Background

Axial spondyloarthritis (SpA) is one of the important causes of inflammatory back pain. Axial SpA involves the axial spine, particularly the sacroiliac (SI) joint and is known to be associated with HLA-B27 [1]. Ankylosing spondylitis (AS), the key disease entity of axial SpA, is the most well known, late form of axial SpA with radiographic change. Of note, detection of sacroiliitis by imaging studies is important in diagnosing AS [2]. Plain radiography has been the mainstay imaging modality for AS and is also essential for evaluating disease progression [3,4]. The early form of axial SpA (early axial SpA) is an axial SpA without plain radiographic change and diagnosed by clinical characteristics or magnetic resonance imaging (MRI). Recently, new treatment option (TNF-α therapy, etc.) for axial SpA necessitate early detection of early axial SpA [5].

Bone scintigraphy also has been used for assessing sacroiliitis. The SI joint to sacrum uptake ratio [sacroiliac joint/sacrum (SIS) ratio] can be measured from the scan: an increased SIS ratio is found mostly in AS patients compared with healthy controls [6,7]. Prominent SI joint uptake and the increased SIS ratio in bone scintigraphy generally portray an advanced stage of sacroiliitis. However, the SIS ratio measured in bone scintigraphy uses a rectangular region of interest that does not represent the accurate uptake of the SI joint and sacrum [8]. Previous studies have tackled the possible limitation of conventional bone scintigraphy in detecting the early manifestations of axial SpA [9-12].

Computed tomography (CT) has been validated for the diagnosis of axial SpA, especially when there are structural changes in the SI joint [13,14]. Recently, magnetic resonance imaging (MRI) has been incorporated as an imaging tool for the new assessment of spondyloarthritis international society (ASAS) classification criteria of axial SpA [15-19]. The ASAS/OMERACT MRI group has recently published definitions of MRI findings in axial SpA. In general, MRI is a useful imaging tool to demonstrate bone marrow edema, osteitis, and enthesitis; however, the cost or accessibility of MRI can become a limiting factor.

In recent years, single positron emission computed tomography (SPECT)/CT has been used in various fields in medicine for functional and anatomical imaging [20]; SPECT/CT increased specificity compared with SPECT in clinical practice by conjoining the anatomical information provided by CT [21,22]. Compared with conventional bone scintigraphy, SPECT/CT can exclude physiological uptake and better characterize equivocal lesions [23]. In the musculoskeletal field, SPECT/CT can help delineate changes of bone adjacent to the synovial surface. Bone SPECT was shown to be superior to bone scintigraphy in measuring the SIS ratio [24]; thus, the fusion of SPECT and CT may further provide useful information in detecting early sacroiliitis [25]. To date, no study exists regarding bone SPECT/CT findings in early axial SpA.

In this study, we investigated the characteristics of bone SPECT/CT imaging with volume of interest (VOI) analysis in early axial SpA patients, comparing the findings with those observed in bone scintigraphy and plain radiography.

## Methods

### Patient selection

Between March 2011 and December 2012, 20 patients with inflammatory back pain (IBP) meeting the Amor criteria for SpA (range: 5–10) yet with minimal or no change in the SI joint in plain radiography were prospectively enrolled at the Seoul Metropolitan Government-Seoul National University Hospital Boramae Medical center (SMG-SNU Boramae Medical center) [26]. Patients met four or more of the following five criteria of IBP: improvement with exercise, pain at night, insidious onset, age at onset < 40 years, and no improvement with rest [27]. Symptom onset had to be less than 5 years. As a control group, 13 individuals with mechanical hip or back pain, no history of trauma or injury in the area of interest, and no abnormal findings in plain radiography were included. The patients involved in our study underwent bone scintigraphy and bone SPECT/CT for purpose of research. Written informed consent for participation in this study was obtained from participants. This study was approved by the Institutional Review Board of SMG-SNU Boramae Medical center.

### Bone scintigraphy and bone SPECT/CT acquisition

Bone scintigraphy was performed 3 hours after injection of 1110 MBq (30 mCi) Tc-99 m-hydroxy-diphosphonate (HDP). Next, we obtained bone SPECT/CT images using the Hawkeye 4 system (GE Healthcare, Milwaukee, WI, USA); low-dose CT and SPECT images were acquired in turn for 10 and 20 min, respectively. CT images were obtained using 5.0 mm slices and 512 × 512 matrices with a standard filter (140 kV, 2.5 mA). SPECT images were acquired using a 1.5X zoom, 128 × 128 matrices, and a step–and-shoot scan mode. Fusion of CT and SPECT images was performed automatically using intrinsic software.

### Analysis of sacroiliac joint lesions

The SIS ratio was measured by both bone scintigraphy and bone SPECT/CT. For bone scintigraphy, rectangular regions of interest (ROIs) were drawn on both SI joints and the sacrum from the posterior planar image. The mean uptake was used to estimate the SIS ratio in bone scintigraphy [7].

**Figure 1:**
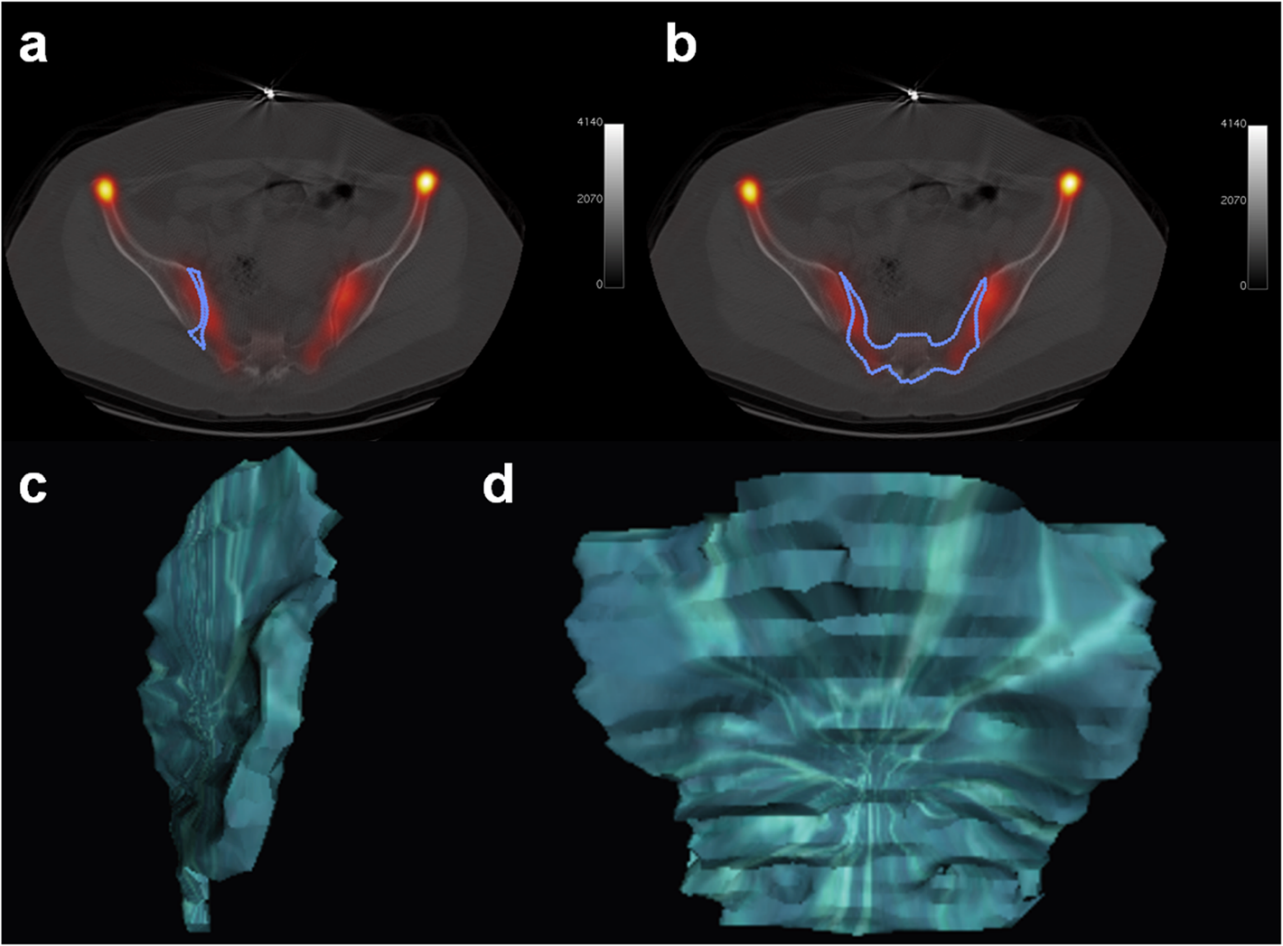
**The volume of interest (VOI) of sacroiliac (SI) joint and sacrum for SI joint to sacrum (SIS) uptake ratio.** The VOI images of SI joint **(a)** and sacrum **(b)** defined in transxial image of bone single positron emission computed tomography-computed tomography (SPECT/CT). Three-dimensional (3D) reconstructed VOI images of SI joint **(c)** and sacrum **(d)** used to calculate the SIS uptake ratio in bone SPECT/CT.

In SPECT/CT, the VOI was used to measure the uptake in both SI joints and the sacrum. VOI was defined using CT images of SPECT/CT. SI joint was defined by the space between sacrum and iliac bone from the start to end of the SI joint. The VOI of the SI joint was defined by the surface of iliac bone and sacrum, the shortest line between the edge of iliac bone and sacrum on each transaxial CT image (Figure 1a). VOI of the sacrum was defined by the bone margin from the start to end of the sacrum on each transaxial CT images (Figure 1b). Each VOI was reconstructed three-dimensionally. After determining the VOI of the SI joint (Figure 1c) and sacrum (Figure 1d), the mean uptake of each VOI in SPECT was used to calculate the SIS ratio. Two individuals performed the VOI measurement of SI joints twice for reproducibility.

In plain radiography, the degree of sacroiliitis was graded according to a 5-point grading system (0 = normal, 1 = suspicious, 2 = sclerosis and some erosions, 3 = severe erosions, widening of the joint space and some ankylosis, 4 = complete ankylosis) [28]. Plain film image was analyzed by two radiologists specialized in musculoskeletal radiology, who were blinded to patient’s clinical findings. The final report with which both radiologists concurred was used in this study.

### Statistical analysis

First, we compared the clinical characteristics and laboratory tests between early axial SpA patients and control subjects. Continuous parameters (age when diagnosis was made, age of symptom onset, symptom duration, body mass index [BMI], erythrocyte sedimentation rate [ESR], C-reactive protein [CRP]) were analyzed by Mann–Whitney *U* test, and categorical parameters (gender ratio, HLA-B27, peripheral arthritis, enthesitis, uveitis, family history of SpA) were studied by Fisher’s exact test. Reproducibility test was performed for bone SPECT/CT VOI analysis using intra-class correlations (ICC) and coefficients of variation (CV). And the SIS ratio of bone scintigraphy and bone SPECT/CT between early axial SpA patients and controls was compared using Mann–Whitney *U* test. Receiver operating characteristic (ROC) curve analysis was performed for bone scintigraphy and bone SPECT/CT, along with area under the curve (AUC), cut-off value, sensitivity, and specificity of each modality. Lastly, Kruskal-Wallis test was used to compare the SIS ratio of bone SPECT/CT among different grades of sacroiliitis in plain radiography. All statistical analyses were lesion based and performed using SPSS software (ver. 18.0; SPSS Inc., Chicago, IL, USA) and MedCalc (MedCalc Inc., Mariakerke, Belgium). A *p*-value less than 0.05 was deemed to indicate statistical significance.

## Results

### Patient characteristics

The mean age of the 20 early axial SpA patients was 35.3 ± 12.5 years (male: female ratio = 12:8, age range = 17–65 years). The mean age of symptom onset was 33.2 ± 13.5 years (age range = 10–65 years), and the mean symptom duration was 24.0 ± 18.4 months (range = 1–60 months). No significant difference was noted in age, age of symptom onset, symptom duration and BMI between axial SpA patients and control subjects. Early axial SpA patients showed significant positive symptoms of HLA-B27 (*p* = 0.002), and peripheral arthritis (*p* = 0.002), and increased ESR (*p* = 0.032) and CRP (*p* = 0.004) compared with control subjects. Other parameters showed no significant change (Table 1).

**Table 1:** **Characteristics of the subjects**

	**Early axial SpA (n =20)**	**Controls (n = 13)**	***p*** **-values**
Age when diagnosis was made (yr)	35.3 ± 12.5	42.5 ± 13.4	0.128
Age of symptom onset (yr)	33.2 ± 13.5	34.2 ± 13.4	0.945
Body mass index (BMI)	24.2 ± 4.5	23.8 ± 1.4	0.905
Gender ratio (M:F)	12:8	4:9	0.157
HLA-B27 (+)	10/20	0/13	0.002*
Peripheral arthritis (+)	10/20	0/13	0.002*
Enthesitis (+)	3/20	0/13	0.261
Uveitis (+)	1/20	0/13	1.000
Family history of SpA (+)	2/20	0/13	0.507
ESR (mm/h)	27.9 ± 7.0	10.5 ± 3.2	0.032*
CRP (mg/L)	1.7 ± 0.6	0.2 ± 0.1	0.004*

SpA = spondyloarthritis; ESR = erythrocyte sedimentation rate; CRP = C-reactive protein.

*Statistically significant.

### Reproducibility of bone SPECT/CT VOI analysis

ICC and %CV of bone SPECT/CT VOI analysis were 0.814 (range = 0.696-0.886, *p* < 0.001) and 5.6%, respectively, which demonstrates reliable reproducibility.

### Comparison of the SIS ratio between early axial SpA patients and control subjects

**Figure 2:**
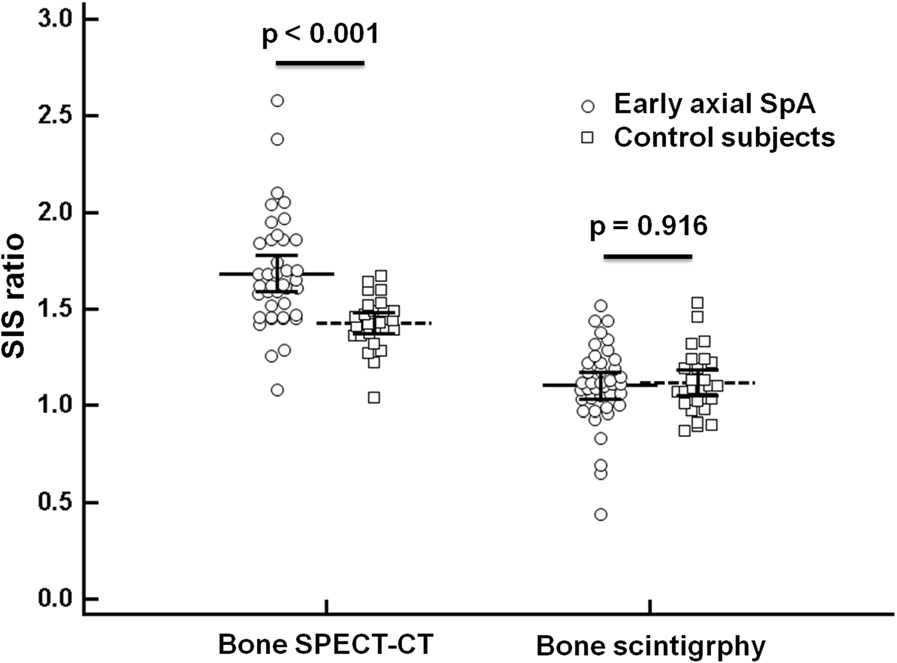
**Comparison of SIS ratio between early axial SpA and control subjects.** A significant difference of SIS ratio was found in early axial SpA patients compared with control subjects by bone SPECT/CT (1.68 ± 0.29 vs. 1.43 ± 0.14, respectively; p < 0.001). However, no significant difference was detected by bone scintigraphy (1.10 ± 0.21 vs. 1.12 ±0.17, respectively; p = 0.916).

**Figure 3:**
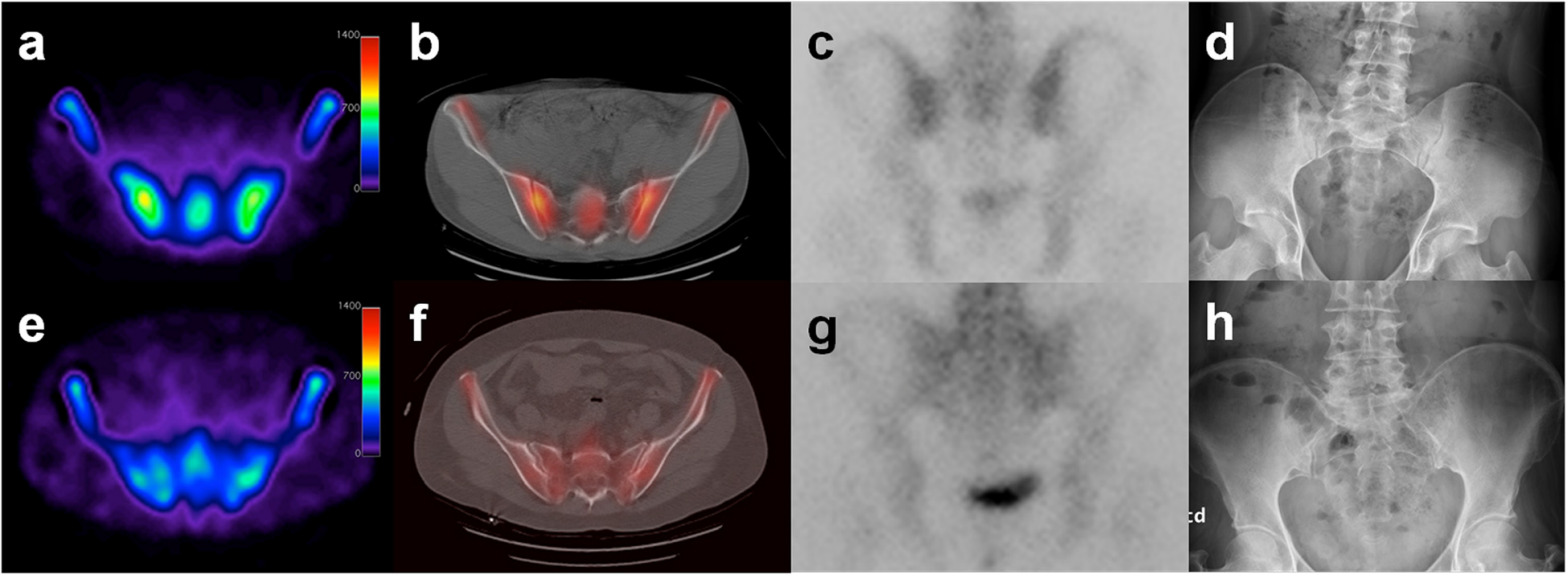
**An example of an early axial spondyloarthritis (SpA) patient (a–d).** Transaxial images of bone SPECT **(a)** and SPECT/CT **(b)** demonstrated uptake in SI joints with high SIS ratios of 1.86 (right) and 1.62 (left). The posterior image of bone scintigraphy **(c)** showed normal SIS ratios of 1.11 (right) and 1.09 (left). The anterior image of plain radiography **(d)** showed SI joint grades of 1 (right) and 0 (left). An example of a control subject **(e–h)**. Transaxial images of bone SPECT **(e)** and SPECT/CT **(f)** showed comparable SIS ratios of 1.04 (right), and 1.22 (left). The posterior image of bone scintigraphy **(g)** showed normal SIS ratios of 1.09 (right) and 0.91 (left). The anterior image of plain radiography **(h)** showed an SI joint grade of 0 (both sides).

A significant increase in the SIS ratio was observed in early axial SpA patients compared with control subjects by bone SPECT/CT (1.68 ± 0.29 *vs*. 1.43 ± 0.14, respectively; *p* < 0.001). However, no significant difference in the SIS ratio was found between early axial SpA patients and control subjects by bone scintigraphy (1.10 ± 0.21 *vs.* 1.12 ± 0.17, respectively; *p* = 0.916) (Figures 2 and 3).

### ROC curve analysis of the SIS ratio in bone SPECT/CT and bone scintigraphy

**Figure 4:**
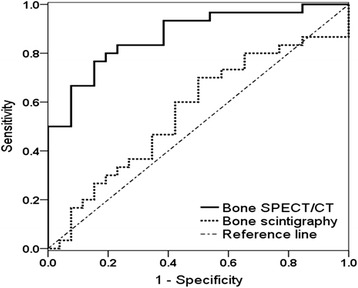
**Receiver operating characteristic (ROC) curve analysis of bone SPECT/CT and bone scintigraphy.** The area under the curve (AUC) of bone SPECT/CT was 0.862 (p < 0.001) and that of bone scintigraphy was 0.523 (p = 0.832). Bone SPECT/CT showed a sensitivity of 80.0% and specificity of 84.6% with a cut-off SIS ratio of 1.50.

ROC curve analysis showed an AUC of 0.862 by bone SPECT/CT [95% confidence interval (CI) = 0.736 – 0.987; *p <* 0.001] and 0.523 by bone scintigraphy (95% CI = 0.310 – 0.737; *p =* 0.832). The ROC curves of the two imaging modalities were significantly different (*p* < 0.001). Bone SPECT/CT revealed a sensitivity of 80.0% and specificity of 84.6% with an SIS ratio cut-off value of 1.50, whereas bone scintigraphy showed a sensitivity of 65.0% and specificity of 53.9% with a cut-off value of 1.08 (Figure 4).

### Analysis of the SIS ratio of bone SPECT/CT according to plain radiography

A total of 40 SI joints of early axial SpA patients were graded by plain radiography. The number of SI joints with a grade of 0, 1, or 2 was 15, 18, or 7, respectively. The SIS ratio of bone SPECT/CT increased in grade 1 or 2 of sacroiliitis than grade 0 of plain radiography (grade 0 = 1.54 ± 0.18, grade 1 = 1.80 ± 0.31, and grade 2 = 1.81 ± 0.19, *p* = 0.007). Statistically significant group difference was found between grade 0 vs. grade 1 and grade 0 vs. grade 2 of plain radiography in post hoc analysis In bone scintigraphy, no significant difference in the SIS ratio among sacroiliitis grades was identified (*p* = 0.065) (Table 2).

**Table 2:** **Analysis of the SIS ratio of bone SPECT/CT according to SI joint grade in plain radiography**

	**Grade 0 (n = 15)**	**Grade 1 (n = 18)**	**Grade 2 (n = 7)**	***p*** **-values**
Bone SPECT/CT	1.54 ± 0.18	1.80 ± 0.31	1.81 ± 0.19	0.007*
Bone scintigraphy	1.00 ± 0.20	1.17 ± 0.18	1.17 ± 0.16	0.065

SIS = sacroiliac joint to sacrum, SPECT/CT = single positron emission computed tomography/computed tomography; SI = sacroiliac.

*Statistically significant group difference exists between grade 0 vs. grade 1 and grade 0 vs. grade 2 of plain radiography in post hoc analysis.

## Discussion

Plain radiography has been the mainstay of SI joint imaging; however, this modality is mainly useful in identifying advanced stages of axial SpA, usually AS. In addition, CT alone is an imperfect imaging modality for diagnosing active sacroiliitis [29]. In the present study, we investigated the usefulness of bone SPECT/CT with VOI analysis for early axial SpA, the early stage of the axial SpA. We demonstrated that bone SPECT/CT is advantageous over conventional bone scintigraphy in detecting sacroiliitis. With the findings of CT, bone SPECT/CT not only can better delineate the anatomy and joint damage but also can identify the volume of the SI joint and sacrum, leading to a better estimation of the SIS ratio. As we used same radiotracer for bone scintigraphy and bone SPECT/CT image acquisition (Tc-99 m-HDP) with one injection of radiotracer, there is no further radiation due to SPECT image acquisition. Furthermore, CT of SPECT/CT scanner of this study is combined low-dose CT which could minimize the radiation exposure by CT to the patient.

Tc-99 m-HDP is specifically absorbed to the bone, and the radiotracer uptake depends on local blood flow and rate of extraction of the bone [30]. The bony changes of early SpA in the axial bone can induce increased Tc-99 m-HDP uptake and can be identified by SPECT/CT. To prove this, we introduced a new method of SIS ratio measurement in SPECT/CT with the mean uptake in a pre-defined volume of the SI joint and sacrum. Previous studies in the nuclear medicine field had some drawbacks in quantifying the SIS ratio due to overlapping of the adjacent structures without a definite demarcation of the bony structure [8]. Some papers have insisted the usefulness of early detection of AS by bone scintigraphy [31,32] or SPECT alone [33]; however, their conclusions are not commonly accepted, and shows limited value for diagnosing probable or suspected AS as well as established AS [8]. SPECT offers more information than planar imaging of the SI joint; however, accurate delineation of the SI joint or sacrum is nearly impossible. SPECT/CT provides much more precise information and resolution of the area of interest than planar imaging obtained by bone scintigraphy and SPECT. Furthermore, using the CT image for delineating the sacrum and SI joint decreased the inter-observer variation.

As our aim was to investigate the difference in the SIS ratio between SPECT/CT and bone scintigraphy in early axial SpA, ROC curve analysis of both bone SPECT/CT and bone scintigraphy was performed to predict the best cut-off value to aid in the diagnosis of early axial SpA. A SIS ratio greater than 1.50 by SPECT/CT demonstrated the best sensitivity (80.0%) and specificity (84.6%). In fact, the optimal cut-off value of the SIS ratio may differ depending on the radiopharmaceutical and imaging modality. For instance, a SIS ratio greater than 1.2 has been commonly observed in patients with sacroiliitis in conventional bone scintigraphy. A recent study showed a SIS ratio cut-off value of 1.3 for F-18-fluoride PET-CT in AS [34]. Another study report that F-18-fluoride PET/CT, the bone turnover radiotracer, was useful to diagnose ankylosing spondylitis, while F-18-FDG PET/CT was not [35]. We hypothesized that Tc-99 m-HDP is also the bone turnover agent like F-18-fluoride, it reflected early SpA well, and expect it can be used with lower cost and availability compared with F-18-fluoride PET/CT.

Notably, SPECT/CT was able to differentiate the subtle changes of sacroiliitis by plain radiography (grade 0 *vs.* grade 1 or 2), in contrast to bone scintigraphy. Previous studies have shown that the SIS ratio in bone scintigraphy is greater in advanced SI joint lesions by plain radiography [36]. However, this finding was only appreciated in sacroiliitis of grade 3 or higher; in other words, AS, the late form of axial SpA [3]. All 20 axial SpA patients in our study met the Amor criteria without evident plain radiography findings (early axial SpA; less than grade 2). Thus, our data indicate that bone SPECT/CT is more useful in identifying early changes in the SI joint compared with conventional bone scintigraphy.

There are several limitations in our study. First, this study consists of relatively small number of early axial SpA patients. As the purpose of our study was to specifically demonstrate the bone SPECT/CT SI joint findings in early SpA patients, we did not obtained clinical data associated with disease activity, such as Bath ankylosing spondylitis disease activity index (BASDAI) or patient’s global assessment. In addition, MRI comparison data for diagnosis of early axial SpA, and predicting and monitoring treatment response using bone SPECT/CT needs to be evaluated in the future [37]. We are planning a prospective comparative study using bone SPECT/CT, F-18-fluoride PET/CT [34] and MRI [38,39] for the diagnosis of early axial SpA with long-term follow up. Lastly, advantages of follow-up imaging with SPECT/CT would be need to be weighed against repeated radiation exposure and should be considered carefully.

## Conclusions

The SIS ratio obtained by bone SPECT/CT, is more useful than conventional bone scintigraphy or plain radiography in diagnosing early axial SpA. Low-dose CT adds structural information and helps detailed VOI information of the SI joint and sacrum. We propose 1.50 as the cut-off SIS ratio to consider for the diagnosis of early axial SpA by bone SPECT/CT.

## Abbreviations

SPECT: Single positron emission computed tomography
CT: Computed tomography
SI: Sacroiliac
VOI: Volume of interest
SpA: Spondyloarthritis
SIS ratio: Ratio between the entire SI joint and sacrum
ESR: Erythrocyte sedimentation rate
CRP: C-reactive protein
ICC: Intra-class correlations
CV: Coefficients of variation
ROC: Reciever operating characteristic
AUC: Area under curve
AS: Ankylosing spondylitis
MRI: Magnetic resonance imaging
ASAS: Assessment of spondyloarthritis international society
HDP: Hydroxy-diphosphonate
BASDAI: Bath’s ankylosing spondylitis disease activity index
